# Southern Med Review to Journal of Pharmaceutical Policy and Practice: broadening the remit!

**DOI:** 10.1186/2052-3211-6-1

**Published:** 2013-06-13

**Authors:** Zaheer-Ud-Din Babar, Shane Scahill, Ayyaz Kiani, Caroline Vaughan

**Affiliations:** 1 School of Pharmacy, University of Auckland, Auckland, New Zealand; 2 Devnet Consultants, Islamabad, Pakistan; 3 Robert Jones Agnes Hunt Orthopaedic Foundation Trust, Oswestry, Shropshire, UK

## 

We are happy to announce the metamorphosis of the *Southern Med Review* (*SMR*) into the *Journal of Pharmaceutical Policy and Practice*[1-3]. As an open access journal published by BioMed Central [4-7], *Journal of Pharmaceutical Policy and Practice* (*JoPPP*) will reach a greater number of readers. Open access will lead to a wider readership, higher downloads, higher citations, and a higher Impact Factor [7-9]. Articles are archived in PubMed Central, and other freely accessible full-text repositories which also extends the access by readership.

While rejoicing the start of an exciting new phase, it seems appropriate to review where we have come from and where are we heading with this new Journal. The first issue (Vol 1 Issue 1) of *SMR* was published on 23rd Dec 2008 [1,2]. Since its beginning, nine issues have been published which include a total of 70 articles. Issues covered have included medicines *access* (governance, development and pharmaceutical systems, pharmacoeconomics, medicine pricing policies, patents and TRIPS, and affordability) as well as medicines *use* (socio behavioural aspects of medicine use, pharmacoepidemiology, pharmacovigilance, drug utilization studies, pharmacy practice issues and concerns) [1].

The main aim of *SMR* was to promote pharmaceutical policy research from low and middle income countries - a job which the journal did well. *SMR* provided opportunity and support to emerging researchers with 30 to 40% of the contributors being first time authors [1]. The journal also published studies on the pharmaceutical situation of countries where little or no pharmaceutical literature is available e.g. Slovenia, Macedonia and Afghanistan [10-12]. *SMR* was commended by the World Health Organisation (WHO) and its policy impact was evident from published studies which were instrumental in changes in the medicines pricing policies of Vietnam and Thailand [13].

Now the journal is entering a new phase and is renamed the *Journal of Pharmaceutical Policy and Practice* (*JoPPP*) [3]. This change in title reflects a broadening in the remit of the Journal. As alluded to above, each word in the title of the new journal has been carefully selected and we have taken the opportunity to outline what these words mean to us and how we see them relating to the scope of *JoPPP*.

*SMR* was an internationally focussed journal with a wide geographic and socioeconomic flavour; although most of the research centred on the developing world [1,2]. Academics residing in both the developed and the developing world generated interesting policy and practice related work; more often policy than practice! We expect that this will continue under the *JoPPP* flagship. However, in line with the broadening remit we expect to receive more pharmaceutical policy related work from those in high-income countries.

The word *Pharmaceutical* was also chosen with care; to be intentionally broad and to differentiate from *Pharmacy* per se. The term pharmacy encompasses the physical structure of the community or hospital pharmacy and the practices within that. However, pharmacies are only one component of a larger complex health system which influences global access to, and use of, medicines. The term pharmaceutical encompasses pharmacy but is broader in that it involves wider aspects of medicines access and use including: technology, the pharmaceutical industry, other health professionals that influence use such as doctors and nurse prescribers and so forth. Pharmaceutical policy includes such areas as national pharmaceutical policy, pharmacoeconomics [14], regulatory affairs, pharmaceutical industry inspection; to name just a few examples. There do however remain many unanswered questions in the field of pharmaceutical policy.

From a practice viewpoint, international policy has been churned out of government departments in high income countries; particularly in the commonwealth [15]. However, there is little understanding of the degree to which these policies are being adhered to and a global systematic review of international medicines policies at the level of the health system as a whole is yet to be undertaken. These are considerable tasks and country and/or continent level reviews of the status of pharmaceutical policy and practices would be useful. Defining what “Pharmaceutical Policy” is globally, and its scope as a concept and instrument for change across a range of country contexts, will be a sensible first step to doing this.

Finally, adding the word ‘practice’ to the *JoPPP* title warrants consideration. There are a number of contexts that encompass pharmaceutical practice and we will be particularly interested in the studies related to community pharmacy setting. This could range from the papers discussing policies and their impact on pharmaceutical practice. Also, the interest would be in medicines compliance, concordance, rationale use of medicines, medicines use among minorities and the disadvantaged groups as well those in the disaster or conflict zones.

We believe pharmaceutical policy is encompassing and directing activities which are related to access to medicines and their use. We look forward to receiving more of these papers as *JoPPP* continues to grow and morph. We encourage our readers to keep their contributions rolling in!
